# UV-Sensitivity of Shiga Toxin-Converting Bacteriophage Virions Φ24_B_, 933W, P22, P27 and P32

**DOI:** 10.3390/toxins7093727

**Published:** 2015-09-21

**Authors:** Sylwia Bloch, Bożena Nejman-Faleńczyk, Gracja Topka, Aleksandra Dydecka, Katarzyna Licznerska, Magdalena Narajczyk, Agnieszka Necel, Alicja Węgrzyn, Grzegorz Węgrzyn

**Affiliations:** 1 Department of Molecular Biology, University of Gdańsk, Wita Stwosza 59, Gdańsk 80-308, Poland; E-Mails: sylwia.bloch@biol.ug.edu.pl (S.B.); bozena.nejman@biol.ug.edu.pl (B.N.-F.); gracja.topka1@wp.pl (G.T.); ola.dydecka@wp.pl (A.D.); katarzyna.licznerska@biol.ug.edu.pl (K.L.); agnieszkanecel@gmail.com (A.N.); 2 Laboratory of Electron Microscopy, University of Gdańsk, Wita Stwosza 59, Gdańsk 80-308, Poland; E-Mail: magdalena.narajczyk@biol.ug.edu.pl; 3 Laboratory of Molecular Biology (affiliated with the University of Gdańsk), Polish Academy of Sciences, Wita Stwosza 59, Gdańsk 80-308, Poland; E-Mail: alicja.wegrzyn@biol.ug.edu.pl

**Keywords:** Shiga toxin-converting bacteriophages, virion stability, UV irradiation

## Abstract

Shiga toxin-converting bacteriophages (Stx phages) are present as prophages in Shiga toxin-producing *Escherichia coli* (STEC) strains. Theses phages can be transmitted to previously non-pathogenic *E. coli* cells making them potential producers of Shiga toxins, as they bear genes for these toxins in their genomes. Therefore, sensitivity of Stx phage virions to various conditions is important in both natural processes of spreading of these viruses and potential prophylactic control of appearance of novel pathogenic *E. coli* strains. In this report we provide evidence that virions of Stx phages are significantly more sensitive to UV irradiation than bacteriophage λ. Following UV irradiation of Stx virions at the dose of 50 J/m^2^, their infectivity dropped by 1–3 log_10_, depending on the kind of phage. Under these conditions, a considerable release of phage DNA from virions was observed, and electron microscopy analyses indicated a large proportion of partially damaged virions. Infection of *E. coli* cells with UV-irradiated Stx phages resulted in significantly decreased levels of expression of *N* and *cro* genes, crucial for lytic development. We conclude that inactivation of Stx virions caused by relatively low dose of UV light is due to damage of capsids that prevents effective infection of the host cells.

## 1. Introduction

Virulence of Shiga toxin-producing *Escherichia coli* (STEC) strains, including their most dangerous subset, enterohemorrhagic *E. coli* (EHEC), depends on production of Shiga toxins [1,2]. These toxins are deleterious to humans due to strong inhibition of protein synthesis, mediated by specific action of the toxin *N*-glycosidase activity which leads to modification and inactivation of rRNA [3,4]. Infections by STEC strains are characterized by high morbidity and mortality, mostly as effects of severe complications, including hemolytic uremic syndrome [5]. The high percentage of fatal cases among infected patients take place not only in geographical regions of low levels of medical care, but also in highly developed countries [6,7,8].

In all STEC strains investigated to date, genes coding for Shiga toxins (*stx* genes) are located on lambdoid prophages, called Shiga toxin-converting prophages or Stx prophages [9]. The Stx bacteriophages belong to the family of lambdoid phages due to similarities of their genomes to that of bacteriophage λ [10]. In lysogenic bacteria, *stx* genes, like vast majority of phage genes, are silent or expressed at low levels [10,11,12,13,14,15]. Prophage induction, caused by any factors or agents that either provoke the bacterial S.O.S. response or weaken the cI repressor binding to phage promoters [13,16], results in excision of phage DNA from the host chromosome and initiation of the lytic development [12]. At this phase, beside expression of genes coding for phage structural proteins and those involved in phage DNA replication, recombination and regulatory processes, *stx* genes are also activated. This leads to effective production of Shiga toxins which are released after phage-mediated lysis of the host cell [9,10].

Since lytic development of Stx phages leads not only to production of Shiga toxins, but also to formation of phage progeny and its liberation after cell lysis, it is clear that newly produced virions can infect neighboring *E. coli* cells. If these cells are not STEC, lysogenization of the new host results in formation of a new STEC strain. Such a scenario is not only theoretical, but it was also documented experimentally [17,18]. In this light, it is important to determine the level of persistence of Stx virions in natural environment, as well as their sensitivity to various factors and agents which could be potentially used for the control of spreading of Stx phages. Both these aspects were investigated previously [19,20,21,22,23,24,25,26], however, while the phenomena were described qualitatively and quantitatively, their specific molecular mechanisms remain largely unknown, particularly at low doses of various factors or agents which correspond to those occurring in the nature. In this work, we have studied sensitivity of virions of five Stx phages to UV irradiation in relation to those of bacteriophage λ, as well as mechanisms of decreased infectivity of Stx virions treated with UV.

## 2. Results and Discussion

**Figure 1:**
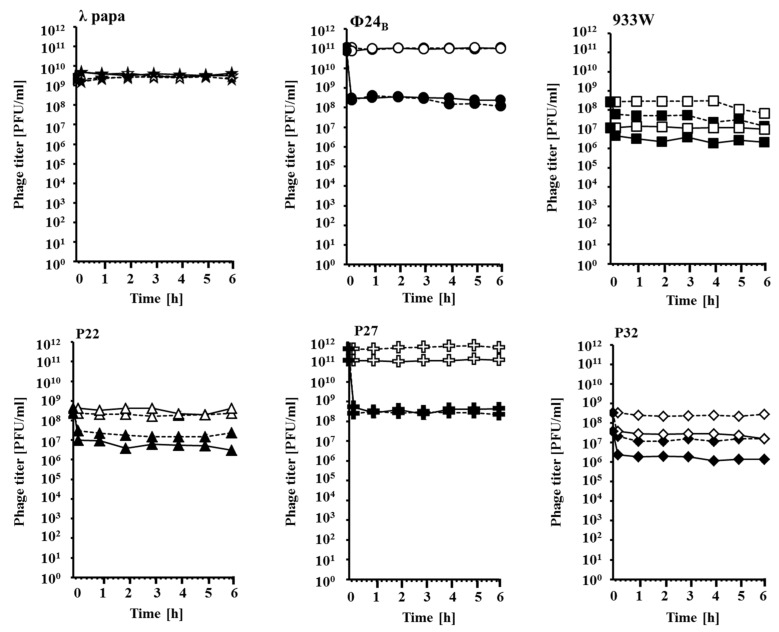
Sensitivity of lamdoid bacteriophages to UV irradiation. Lysates of phages (indicated in each panel) were either non-irradiated (open symbols) or UV-irradiated at 50 J/m^2^ (closed symbols), and *E. coli* MG1655 cells were infected at 37 °C (solid lines) or 43 °C (dashed lines). Phage titer was determined at indicated times after irradiation by estimating number of plaque forming units (pfu) per mL. Results are presented as mean values from three independent experiments with error bars indicating SD, however the bars are smaller than the size of symbols. For all experiments but those with bacteriophage λ, statistically significant differences (*p* < 0.05 in the *t-*tets) between irradiated and non-irradiated phages were found. The use of phage lysates of different initial titers gave very similar results, thus, the sensitivity to UV irradiation was independent on virion density.

Since the influence of temperature on UV-stimulated spreading of Stx phages has been reported previously [18], we have tested effects of UV irradiation on lambdoid virions at 37 °C (the physiological temperature for *E. coli*, a host for these phages) and 43 °C (an elevated temperature which might simulate conditions occurring in an infected mammalian organism). When a lysate of bacteriophage λ was irradiated with UV light at the dose of 50 J/m^2^, no effects on infectivity of virions could be observed at both tested temperatures, 37 °C and 43 °C (Figure 1). However, UV irradiation of virions of five different Shiga toxin-converting bacteriophages (Stx phages) resulted in a significant decrease of the number or plaque forming units (pfu) just after irradiation, and this effect was stable to the end of the experiment, *i.e.*, for 6 h after irradiation (Figure 1). The effect was strongly pronounced in phages Φ24_B_ and P27, where the infectivity dropped by two to three orders of magnitude, while in other tested Stx phages (933W, P22 and P32) number of pfu decreased by one order of magnitude or less, though the decrease was still statistically significant (Figure 1). Therefore, we conclude that virions of Stx phages are sensitive to low doses of UV irradiation, contrary to those of bacteriophage λ. This conclusion has been corroborated by measurement of survival of *E. coli* cells infected with UV-irradiated and non-irradiated bacteriophages. While no significant differences were observed in the fraction of bacteria surviving both kinds of infection with bacteriophage λ, significantly more cells survived infection with UV-irradiated Stx phages relative to non-treated counterparts (Table 1). Again, the most spectacular differences were observed in phages Φ24_B_ and P27 (Table 1).

**Table 1:** Survival of *E. coli* MG1655 cells in the liquid culture at 37 °C following infection with lambdoid bacteriophages, either non-irradiated or UV-irradiated, at m.o.i. = 5.

Bacteriophage	Survival of Cells in Infected Culture (% of Survivors)	
	No UV	50 J/m^2^ UV
λ papa	31 ± 6	30 ± 6
Φ24_B_	36 ± 8	88 ± 7 *
933W	34 ± 1	39 ± 2
P22	44 ± 7	68 ± 6 *
P27	43 ± 2	93 ± 1 *
P32	37 ± 7	63 ± 9 *

Asterisks (*) indicate statistically significant differences (*p* < 0.05 in the *t*-test) between results obtained with and without UV.

**Figure 2:**
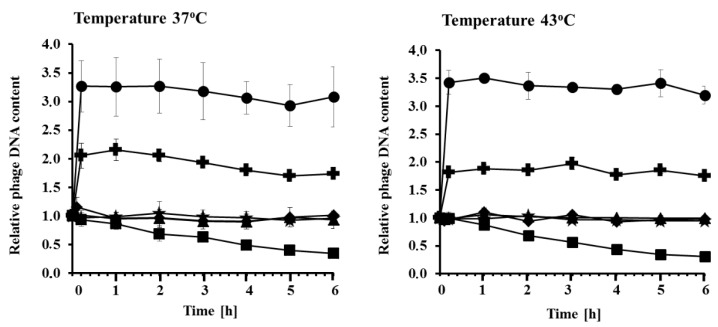
Release of phage DNA from capsids after UV irradiation of virions of lambdoid bacteriophages. Lysates of bacteriophage λ (asterisks) and Stx phages Φ24_B_ (circles), 933W (squares), P22 (triangles), P27 (crosses), and P32 (diamonds) were irradiated with UV light at 50 J/m^2^ at time 0, and incubated at 37 °C or 43 °C as indicated. Amount of released DNA was estimated using Qubit DNA Assay Kit. The results obtained at time 0 are referred to as 1, and other values reflect this value. The presented results are mean values from three experiments with error bars indicating SD (note that in most cases the bars are smaller than the size of symbols).

The results presented in Figure 1 and Table 1 raised a question about the mechanism of the decrease in infectivity of virions irradiated with UV light. Damage of DNA appeared an unlikely reason for this effect, as no significant effects of UV irradiation at 50 J/m^2^ were observed in bacteriophage λ, and there is no reason to suspect significant differences between sensitivity of phages λ and Stx DNAs to UV. Therefore, an alternative hypothesis has been tested, assuming that capsids of Stx virions might be damaged under these conditions. We suspected that severe damage of bacteriophage capsid may result in the release of viral DNA. Therefore, we have determined concentrations of phage DNA in samples of lysates, either untreated or UV irradiated at 50 J/m^2^, by using a fluorescence assay in which a fluorescent dye emits light only when bound to the target molecule. In these experiments, phage DNA included in the capsid is protected from the dye, and thus undetected, while released DNA can be bound to the dye and visualized by fluorescence. We found that while UV irradiation of λ, 933W, P22, and P32 virions resulted in no increase of the released phage DNA amount (the basal level derived from mechanically damaged virions, always present in each lysate), a significant increase in the level of free phage DNA could be observed in experiments with Φ24_B_ and P27 virions (Figure 2). Note that under these experimental conditions, only severe virion damage, allowing to release of the phage DNA from capsids, might be detected. Therefore, since the effects were most pronounced in the same phages as in the measurement of infectivity (Φ24_B_ and P27), we recognized the results shown in Figure 2 as compatible with those presented in Figure 1 and Table 1. In phages 933W, P22 and P32, the UV-mediated damage of virions might be large enough for the loss of infectivity, but too small to fully open the capsid and allow phage DNA to release. Again, no effects of temperature could be detected (Figure 2). One can assume that changes in a capsid may affect infectivity of virions due to either leakiness of phage DNA or lesions in the protein(s) responsible for effective adsorption of the virus on the host cells, or both.

**Figure 3:**
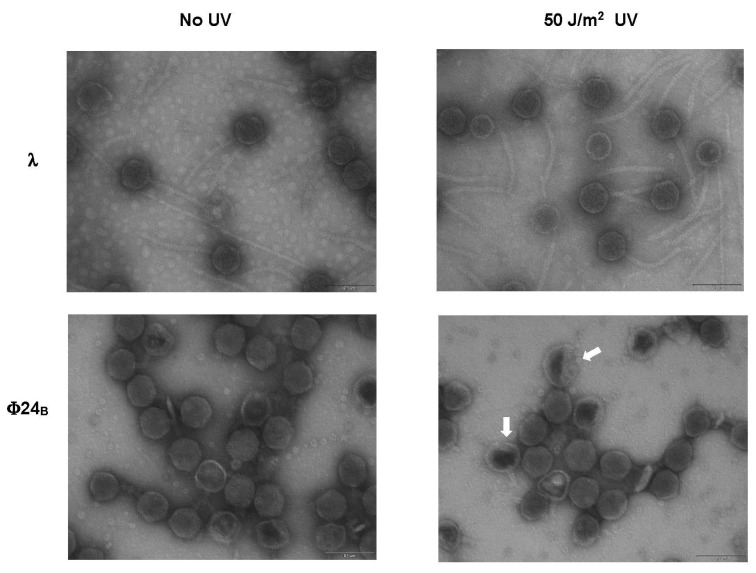
Electron micrographs of virions of bacteriophage λ (upper panels) and Stx phage Φ24_B_ (lower panels), either non-irradiated (left panels) or irradiated with UV light at 50 J/m^2^ (right panels). Untypical Φ24_B_ virions with partially damaged heads are indicated by arrows in the lower right panel. Bars, shown at the lower right corner of each panel, correspond to 100 nm.

To identify what kind of severe damage of Stx virions can be caused by UV irradiation, we have performed electron microscopic studies. Highly purified (with the cesium chloride ultracentrifugation method) virions were UV irradiated (or not, in control experiments) and subjected to electron microscopic analyses. In the vast majority of lysates of tailed bacteriophages, apart from normal virions, those with empty (devoid of DNA) capsids occur at the levels from a few to several percent [27,28]; such “empty head” virions are called “phage ghosts” [29]. The “empty virions” may appear due to defects in virion assembly or as effects of some environmental conditions. For example, osmotic shock causes a significant increase in the fraction of phage ghosts in virion samples as a result of capsid leakiness and release of phage DNA [29]. We have chosen phages λ and Φ24_B_, in which no detectable and the most pronounced loss of infectivity and capsid damage were observed, respectively, for electron microscopic studies. In phage λ, UV irradiation at 50 J/m^2^ resulted in only a slight increase in the fraction of phage ghosts, with no other structural changes detected (Figure 3, Table 2). However, when Φ24_B_ virions were UV irradiated, the fraction of phage ghosts increased considerably relative to non-irradiated samples, and a fraction of untypical virions, with partially destroyed heads, appeared (Figure 3, Table 2). Therefore, we conclude that UV irradiation at the dose of 50 J/m^2^ caused considerable changes in the structure of Φ24_B_ capsid in a significant fraction of virions.

**Table 2:** Summary of the electron microscopic analysis of λ and Φ24_B_ virions, either non-irradiated or UV-irradiated at 50 J/m^2^.

Bacteriophage and Conditions	Fractions of Different Kinds of Virions (%)		
	Normal	Phage Ghosts	Untypical, with Partially Damaged Heads
λ papa (non-irradiated)	93	7	0
λ papa (UV-irradiated)	89	11	0
Φ24_B_ (non-irradiated)	91	9	0
Φ24_B_ (UV-irradiated)	71	23	6

**Figure 4:**
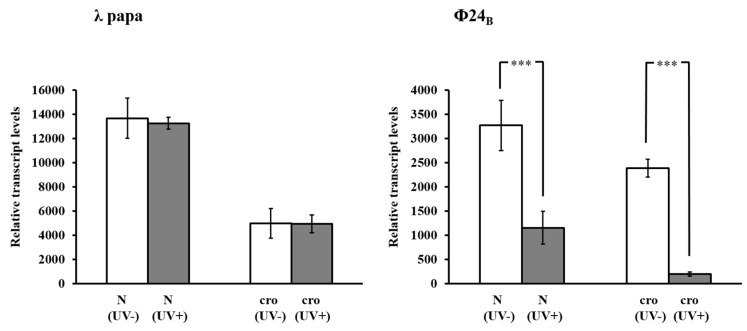
Expression of *N* and *cro* genes of bacteriophages λ (left panel) and Stx phage Φ24_B_ (right panel) which were either non-irradiated (UV−) or irradiated with UV light at 50 J/m^2^ (UV+) prior to infection (at m.o.i. = 1). The mRNA levels were estimated in *E. coli* MG1655 host grown at 37 °C, at 7.5 min or 15 min after infection by λ or Φ24_B_, respectively. The presented results are mean values from three experiments with error bars indicating SD. Statistically significant differences between results obtained for non-irradiated and UV-irradiated phages (*p* < 0.001 in the *t*-test) are indicated by three asterisks.

If our conclusion that decreased infectivity of UV-irradiated Stx phages, relative to untreated ones, results from a damage of capsids in a significant proportion of virions is true, one should expect that lower number of phages can introduce their DNA into host cells, and thus, level of expression of phage genes in a population of infected cells should be considerably lower than in *E. coli* infected with equal number of untreated phages. To test this hypothesis, a sample of the lysate of either λ or Φ24_B_ virions was divided to two halves of equal titer (determined as pfu/mL). One half was UV irradiated at 50 J/m^2^ while the second was not, and then *E. coli* cells were infected with either treated or untreated phages. Following infection, total RNA was isolated, and expression of *N* and *cro* genes (transcribed from the *p*_L_ and *p*_R_ promoter, respectively), crucial for the lytic development [11,12,13,14], was estimated by reverse transcription real-time quantitative PCR (RT-qPCR). Analysis of phage gene expression (RNA level) instead of their copy numbers (DNA level) allowed to be devoid of signal becoming from a fraction of DNA released from capsids in response to UV exposure. In bacteriophage λ, UV irradiation of virions prior to infection did not result in any significant changes in expression of *N* and *cro* genes in the population of *E. coli* cells (Figure 4). On the contrary, in the *E. coli* population infected with irradiated Φ24_B_ bacteriophage, the level of expression of both genes was significantly lower relative to bacteria infected with non-irradiated viruses (Figure 4).

All the results presented in this report indicate that Stx virions are more sensitive to UV irradiation than those of bacteriophage λ. The process of inactivation of bacteriophages with UV light is a known phenomenon, however, UV doses used by others to decrease infectivity of various phages, including Stx phages, were generally significantly higher than those employed in this work [30,31,32,33,34,35]. When high UV doses were employed, inactivation of bacteriophages was ascribed to DNA lesions caused by the irradiation [36]. However, in this study, at the dose of 50 J/m^2^, no inactivation of the model virus, bacteriophage λ, could be detected. Therefore, severe DNA damage is unlikely under these conditions, and other mechanisms had to be considered. The release of phage DNA from capsids (determined biochemically), as well as virion dysmorphology (identified in electron microscopic studies), indicated that damage of virions might be responsible for high UV sensitivity of Stx phages. This may have implications for the spreading of these phages and formation of new STEC strains. Although these processes might be effective in habitats devoid of UV, like in human or animal digestive tracts, one can expect their lower efficiency in other environments, outside of mammalian organism. It remains to be elucidated what structural properties of Stx virions make them labile upon UV irradiation at doses which do not cause any considerable damage of bacteriophage λ capsids.

## 3. Experimental Section

### 3.1. Bacteria and Bacteriophages

*Escherichia coli* MG1655 strain [37] and bacteriophages: λ papa (from our collection), Φ24_B_(∆*stx2*::*cat*) [38], 933W∆tox(∆*stx2*::*catGFP*), 22∆tox(∆*stx2*::*catGFP*), 27∆tox(∆*stx2*::*catGFP*), and 32∆tox(∆*stx2*::*catGFP*), referred to as Φ24_B_, 933W, P22, P27 and P32, respectively, in the text, were used in this work. Bacteriophages Φ24_B_, 933W, P22, P27 and P32 are Shiga toxin-converting phages. Φ24_B_ and 933W have been used for many years as model phages from this group, while P22, P27 and P32 have been described as natural isolates [39]. Derivatives of the Shiga toxin-converting phages 933W, 22, 27, and 32 in which genomes the toxin genes were replaced by *cat* and/or *GFP* markers were described previously [39]. Bacteria were routinely cultured in the Luria-Bertani (LB) medium supplemented with 20 μg/mL chloramphenicol (if required) at 37 °C or 43 °C under aerobic conditions. Bacteriophage suspensions were stored in the TM buffer (10 mM Tris-HCl, 10 mM MgSO4, pH 7.2) (Sigma Aldrich, St. Louis, MO, USA) at 4 °C.

### 3.2. Evaluation of Bacterial Survival after Bacteriophage Infection

The percentage of survived cells after bacteriophage infection was estimated according to the protocol described in details previously [40], with slight modifications. Briefly, host bacteria were grown in LB medium at 37 °C to A_600_ = 0.2. Sample of 1 mL was withdrawn and centrifuged (2000× *g*, 10 min, 4 °C). The pellet was suspended in 1 mL of 0.85% NaCl. Then, the sample was centrifuged again and the pellet was suspended in 1.2 mL of LB medium supplemented with MgSO_4_ and CaCl_2_ (to a final concentration of 10 mM each). Following incubation for 30 min at 37 °C, the volume of analyzed bacteriophage lysate, corresponding to m.o.i. of 5, untreated or treated with UV light (employing the UV lamp provided by Vilber Lourmat, Marne-la-Vallée, France), at 50 J/m^2^, (the dose used routinely for lambdoid prophage induction [41,42]) was added, and then mixture was incubated at 37 °C for additional 30 min. Next, TM buffer (10 mM Tris–HCl, 10 mM MgSO4; pH 7.2) was used to prepare serial dilutions. The volume of 40 µL of each dilution was spread on LB agar plates prior to overnight incubation at 37 °C. Percentage of surviving bacteria was calculated relative to the control sample in which TM buffer was added instead of bacteriophage lysate. Each experiment was repeated three times.

### 3.3. Estimation of Phage Lysate Stability

Phage lysates were obtained after induction of the prophage from the host (*E. coli* MG1655 strain) as described previously [41], with minor modifications. Briefly, lysogenic bacteria were grown in LB medium at 37 °C, in shake flasks with agitation to an A_600_ of 0.1. Prophage induction was provoked by mitomycin C which was added to final concentration of 1 μg/mL. After induction of phage lytic development, the culture was incubated for additional 6 to 10 h. Following 15 min agitation with chloroform (added in the volume of 600 μL per 10 mL), the culture was centrifuged (2000× *g* for 10 min at 4 °C) to remove bacterial debris, and supernatant was filtered using 0.22-μm-pore-size filters (Sigma Aldrich, St. Louis, MO, USA ). Effect of UV irradiation on the phage lysates stability was determined simultaneously at 37 °C or 43 °C, using phage titration method performed according to the standard double agar overlay technique. At times 0 (before UV exposure) and 0.2, 1, 2, 3, 4, 5, 6 h after UV irradiation at 50 J/m^2^, samples of 0.5 mL were withdrawn, and prepared and titrated as described previously [42]. The phage titer, expressed as plaque forming units (PFU) per mL, was determined. For each phage lysate, three replicates were performed.

### 3.4. Measurement of Relative DNA Contents

Phage lysates were prepared as described above. Following UV exposure (50 J/m^2^), one half of the phage lysate was incubated at 37 °C and the second half at 43 °C. Effect of UV irradiation on the amount of DNA released from capsids was determined simultaneously at both temperatures. Before (time zero) and after (the remaining times) exposure of phage lysate to UV light, DNA was quantified by staining with Qubit^®^ dsDNA BR Assay Kit (Invitrogen/Thermo Fisher Scientific, Waltham, MA USA), according to the manufacturer’s protocol. At indicated time points after UV exposure, DNA contents were calculated relative to the DNA amount obtained at time zero which represented value of 1. Each experiment was repeated three times.

### 3.5. Electron Microscopy

Virions were purified from phage lysates obtained after induction of the prophage from the host *E. coli* MG1655 strain with mitomycin C, as described above. Purification was performed using cesium chloride density gradient centrifugation method [43]. Electron microscopic analyses of purified phage particles (untreated and treated with UV light at 50 J/m^2^) were performed employing the Philips CM 100 electron microscope (Philips, Eindhoven, The Netherlands), by using negative staining with uranyl acetate, as described earlier [44].

### 3.6. Bacteriophage Infection and cDNA Sample Preparation

The experiments were performed according to the protocol described previously [45], with slight modifications. Briefly, *E. coli* bacteria were cultured to A_600_ of 0.3 at 37 °C. Then, culture volume of 60 mL was centrifuged and the pallet was washed with 30 mL of 0.85% NaCl. Following additional centrifugation, the sample was suspended in 15 mL of LB medium enriched with MgSO_4_ and CaCl_2_ (to a final concentration of 10 mM each). After incubation at 37 °C for 30 min, the sample was chilled on ice. The appropriate volume of analyzed bacteriophage lysate, corresponding to m.o.i of 1 (treated or untreated with UV light at 50 J/m^2^) was added, and the mixture was incubated on ice. Next, infected bacterial cells were aerated in a water bath shaker at 37 °C. At indicated times (7.5 min for λ and 15 min for Φ24_B_ after phage infection as these phages have various development rates [45]), 1 × 10^9^ cells were treated with NaN_3_ (Sigma-Aldrich, St. Louis, MO, USA) at final concentration of 10 mM, and harvested. The RNA and cDNA samples for real-time PCR assay were prepared as described previously [40,46] using the High Pure RNA Isolation Kit and Transcriptor Reverse Transcriptase (Roche Applied Science, Basel, Switzerland).

### 3.7. Real-Time PCR Assay and Data Analysis

The level of *N* and *cro* phage genes expression was determined by quantitative real-time reverse transcription-PCR (RT-qPCR) using the LightCycler^®^ 480 Real-Time PCR System (Roche Applied Science) and cDNA samples obtained during bacteriophage infection experiment. According to a procedure described previously [47], transcription rates of tested phage genes were compared in parallel to the *icdA* bacterial housekeeping gene. Expression of this gene was found to be stable also upon λ or Φ24_B_ phage infection of *E. coli* MG1655 strain [40,45]. The gene expression analyses were performed using LightCycler^®^ 480 SYBR Green I Master and following primers: pF_λ_N (5′-CTC GTG ATT TCG GTT TGC GA); pR_λ_N (5′-AAG CAG CAA ATC CCC TGT TG); pF_λ_cro (5′-ATG CGG AAG AGG TAA AGC CC); pR_λ_cro (5′-TGG AAT GTG TAA GAG CGG GG); pF_Φ24B_N (5′-AGG CGT TTC GTG AGT ACC TT); pR_Φ24B_N (5′-TTA CAC CGC CCT ACT CTA AGC); pF_Φ24B_cro (5′-CGA AGG CTT GTG GAG TTA GC); pR_ Φ24B_cro (5′-GTC TTA GGG AGG AAG CCG TT); pF_icdA (5′-CGA AGC GGC TGA CCT TAA TTG) and pR_icdA (5′-GTT ACG GTT TTC GCG TTG AT). The reaction components and amplification program were exactly the same as described previously [45]. No template control was included with each run. Gel electrophoresis and melting curve analysis were performed to evaluate the specificity of every amplification reaction. Each experiment was conducted in triplicate. The relative changes in gene expression revealed by quantitative Real-Time PCR experiments were analyzed using the E-Method (with efficiency correction) as described in detail previously [40,45,46].
